# Genomic Diversity and Virulence Factors of *Clostridium perfringens* Isolated from Healthy and Necrotic Enteritis-Affected Broiler Chicken Farms in Quebec Province

**DOI:** 10.3390/microorganisms12122624

**Published:** 2024-12-18

**Authors:** Sara Heidarpanah, Kevin Li, Alexandre Thibodeau, Ilhem Meniaï, Valeria R. Parreira, Sylvain Quessy, Mariela Segura, Nahuel Fittipaldi, Marie-Lou Gaucher

**Affiliations:** 1 Chaire de Recherche en Salubrité des Viandes (CRSV), Département de Pathologie et Microbiologie, Faculté de Médecine Vétérinaire, Université de Montréal, Saint-Hyacinthe, QC J2S 2M2, Canada; sara.heidarpanah@umontreal.ca (S.H.); alexandre.thibodeau@umontreal.ca (A.T.); imeniai@hotmail.com (I.M.); sylvain.quessy@umontreal.ca (S.Q.); 2 Swine and Poultry Infectious Diseases Research Centre (CRIPA), Faculté de Médecine Vétérinaire, Université de Montréal, Saint-Hyacinthe, QC J2S 2M2, Canada; kevin.li.2@umontreal.ca (K.L.); mariela.segura@umontreal.ca (M.S.); n.fittipaldi@umontreal.ca (N.F.); 3 Département de Pathologie et Microbiologie, Faculté de Médecine Vétérinaire, Université de Montréal, Saint-Hyacinthe, QC J2S 2M2, Canada; 4 Groupe de Recherche sur les Maladies Infectieuses en Production Animale (GREMIP), Faculté de Médecine Vétérinaire, Université de Montréal, Saint-Hyacinthe, QC J2S 2M2, Canada; 5 Canadian Research Institute for Food Safety (CRIFS), Food Science Department, University of Guelph, Guelph, ON N1G 2W1, Canada; vparreir@uoguelph.ca

**Keywords:** *Clostridium perfringens*, necrotic enteritis, broiler chickens, Quebec, whole genome sequencing, comparative genomics, antibiotic resistance genes, prophage sequences, pangenome

## Abstract

Avian necrotic enteritis due to the Gram-positive bacterium *Clostridium perfringens* has re-emerged following the ban on antibiotic growth promoters in many poultry producing countries. The limited number of previous studies has left important gaps in our understanding of the genetic diversity and virulence traits of the pathogen. To address these knowledge gaps, in this study, we sequenced the genomes of 41 *Clostridium perfringens* isolates recovered from commercial broiler chicken flocks in Quebec, Canada, including isolates from healthy birds and those affected by necrotic enteritis. We sought to understand the pangenome diversity and interrogated the genomes for key virulence factors involved in necrotic enteritis pathogenesis. On average, the genomes had a GC content of 28% and contained 3206 coding sequences. A variable presence of toxins, degradative hydrolytic enzymes, and collagen-binding proteins was also found. Through pangenome analysis, we revealed a total of 10,223 genes, 652 (6.4%) of which formed the core genome. Additionally, we identified 17 different plasmids, 12 antibiotic resistance genes, and nine prophage regions. Overall, our results demonstrated a relatively high genetic diversity among chicken *Clostridium perfringens* isolates collected from the same geographical location, offering new insights into potential virulence mechanisms and adaptation of the pathogen within poultry populations.

## 1. Introduction

*Clostridium perfringens* is a Gram-positive, spore-forming, rod-shaped, anaerobic bacterium found in diverse environments, such as soil, feces, food, and sewage, in addition to being recognized as a typical member of the gut microbiota in both humans and various animal species [1]. However, certain *C. perfringens* strains possessing an array of different virulence factors can cause a broad range of potentially fatal diseases, including gas gangrene and food poisoning in humans, enterotoxemia in sheep and goats, hemorrhagic gastroenteritis in horses and dogs, and necrotic enteritis (NE) in poultry [2]. NE is a multifactorial gastrointestinal disease affecting commercial broiler chicken flocks and has been well-controlled for several decades using in-feed antibiotics. However, with the important shift taken by the poultry industry in many countries to eliminate antibiotic growth promoters or to limit the use of some categories of antibiotics in animal feed, this gastrointestinal disease now imposes a growing economic burden on the global poultry industry, with a fully effective control strategy still to be developed [3].

Effective control of the disease requires a thorough understanding of its pathogenesis, which would not be complete without an in-depth knowledge of the genetics and virulence attributes of its causative agent [3,4]. To date, the genetic diversity of *C. perfringens* of poultry origin has been investigated using different techniques, including pulsed-field gel electrophoresis (PFGE), ribotyping, multilocus sequence typing, multiple-locus variable number tandem-repeat analysis, microarray comparative genomic hybridization (CGH), and more recently, whole genome sequencing (WGS) [5,6,7,8,9,10,11,12].

Genome analysis of pathogenic *C. perfringens* strains has shown a relatively high genetic diversity, with some toxin-encoding genes, plasmids, and chromosomal regions being strongly associated with NE [9,10,13,14]. In that respect, Lepp et al. used genome sequencing and comparative genomics approaches to identify specific DNA sequences among multiple NE-causing *C. perfringens* [14]. They identified three highly conserved NE-associated loci named NELoc-1 (42 kb), NELoc-2 (11.2 kb), and NELoc-3 (5.6 kb) [14]. Their findings showed that the gene encoding the NetB toxin (*netB*), a virulence factor recognized until recently as crucial for initiating NE, is located on the NELoc-1, which in turn is found on a large conjugative plasmid (pNetB) in NE-causing *C. perfringens* strains [14,15]. Besides *netB*, NELoc-1 also contains 36 additional genes, including those predicted to encode cell surface-associated or extracellular proteins, one putative sortase, and two putative leukocidins, likely involved in *C. perfringens* virulence [14]. Similarly, the NELoc-3 locus is located on a different plasmid that also carries the gene encoding the *C. perfringens* beta2 toxin (*cpb2*). NELoc-2, however, is the only locus found on the bacterial chromosome [14].

A different study by the same research group examined the plasmid profile of 26 isolates recovered from NE-affected and healthy broiler chicken flocks using PFGE [13]. The study revealed that most NE-causing isolates possessed at least three large plasmids, which were both larger and more numerous than plasmids found in *netB*-negative isolates from healthy broiler chicken flocks. Sequencing of two different *netB*- and *cpb2*-positive plasmids (pNetB-NE10 and pCpb2-CP1, respectively) showed the presence of intact *tcp* conjugative regions, suggesting that both plasmids were capable of conjugation [13].

Microarray CGH was also employed to assess the genome content of 63 *C. perfringens* isolates [10]. Results showed that a total of 400 genes were variably present among 54 chicken-derived isolates (both healthy and NE-affected birds) and nine non-poultry isolates. These genes encoded factors with putative functions in carbohydrate metabolism, as well as in capsule and cell wall biosynthesis. The variable genes identified were grouped into 142 genomic regions, 49 of which consisted of genes that were significantly associated with *netB*-positive isolates. These regions included the three previously identified NE-associated loci (NELoc-1–3), as well as loci comprising genes related to adhesion (VR-10B), iron acquisition (VR-22), carbohydrate utilization (VR-05), and plasmid maintenance [10].

When investigating the chromosomal variations among a diverse collection of poultry-derived *C. perfringens* isolates recovered from both healthy and NE-diseased birds, Lacey et al. revealed that the pathogenicity of the bacterium was not associated with the core genome content but rather linked to the accessory genes found on both plasmids and the chromosome [9]. In addition, the authors identified several chromosomal variations between NE pathogenic strains, including the VR-10B adhesion-associated locus previously reported by Lepp et al., capsular polysaccharide synthesis loci, and prophage-like regions, with potential roles of these in the virulence of different NetB-producing NE-causing strains of *C. perfringens* [9]. Results of this study and from others suggested that the presence of diverse prophage elements in the chromosome of *C. perfringens* not only contributes to the variation seen between *C. perfringens* genomes but also represents an important source of virulence determinants, such as the bacteriocin perfrin-encoding gene, which plays a role in the typical single strain dominance of perfrin-expressing *C. perfringens* isolates in NE cases [9,16,17,18]. Furthermore, it has been shown that prophage-like regions affect the sporulation and metabolism of *C. perfringens* through integration into genes involved in these mechanisms [9,10,18].

*C. perfringens* has long been recognized as the causative agent of various histotoxic and enterotoxemic diseases with toxin production being a hallmark of its pathogenesis. In recent years, greater attention has been paid to the role of these toxins in damaging the intestine during NE pathogenesis [2,4]. However, several studies suggest that toxin activity alone is insufficient for *C. perfringens* to cause NE, and other bacterial factors are believed to play essential roles in both the pathogenesis and development of a protective immune response. This has brought particular attention to the colonization ability of avian *C. perfringens* [2,4,15,19,20].

Despite recent advances, many questions about *C. perfringens* pathogenesis remain unanswered, including the extent of the genetic diversity of the organism. In a recent study, some of us used a comparative and subtractive reverse vaccinology approach on isolates from healthy and NE-affected broilers to identify proteins unique to virulent *C. perfringens*, with potential roles in colonization [20]. Building on this previous work, here, through a comparative analysis of 41 *C. perfringens* genomes, including 26 isolates from NE-affected and 15 isolates from healthy broiler chicken flocks in Quebec, Canada, we aimed to further investigate the genetic diversity and catalogue the virulence arsenal of this pathogen.

## 2. Materials and Methods

### 2.1. Sample Collection and Isolation of C. perfringens Strains

*C. perfringens* isolates were previously recovered from fecal samples of healthy and NE-affected commercial broiler chicken flocks in Quebec, Canada [11]. Perfringens Agar Base medium (LAB M, Potters Bar, UK) following sample enrichment in Cooked Meat broth (Oxoid Ltd., Basingstoke, UK) were used to isolate the bacteria and subsequent confirmation by the reverse CAMP test [11]. The recovered isolates were preserved in *Brucella* broth (Becton, Dickinson and Company, Franklin Lakes, MD, USA) containing 30% glycerol (Fisher Scientific, Fair Lawn, NJ, USA) and stored at −80 °C until further analysis [11].

### 2.2. C. perfringens Culture, DNA Extraction, Sequencing Library Preparation, and WGS

The frozen bacterial isolates were grown overnight on 5% sheep blood agar plates (Fisher Scientific, Ottawa, ON, Canada) at 37 °C under anaerobic conditions (Oxoid AnaeroGen gas packs (Thermo Fisher Scientific, Waltham, MA, USA)). Genomic DNA was extracted from the pure cell cultures of *C. perfringens* using the DNeasy Blood and Tissue Kit (Qiagen, Hilden, Germany) as previously described [20]. DNA sequencing libraries were prepared using the Nextera™ XT DNA Library Preparation Kit (Illumina, San Diego, CA, USA) according to the manufacturer’s instructions and sequenced as paired end (250 bp + 250 bp) on an Illumina MiSeq instrument at the facilities of Agriculture and Food Laboratory of the University of Guelph (Guelph, ON, Canada) [20]. Data have been deposited in the NCBI database under the BioProject accession numbers PRJNA734442 and PRJNA1188553.

### 2.3. Bioinformatics Workflow for Genome Assembly and Annotation

Raw reads were de novo assembled into contigs using the A5-miseq pipeline v. 20160825 [21]. The resulting contigs were then joined via the linker sequence NNNNNCACACACTTAATTAATTAAGTGTGTGNNNNN, introducing stop codons in all six reading frames. Genome annotations were performed using the Prokka tool v. 1.14.6 [22] and a custom protein database derived from several previously annotated *C. perfringens* genomes (see Supplementary Table S1).

### 2.4. In Silico Profiling of Virulence Factors

The presence of virulence factors in the newly generated genomes was investigated using BLASTn [23] and a custom database of *C. perfringens* virulence factors, which included seven toxin-encoding genes (*cpa*, *cpb2*, *netB*, *tpeL*, *lam*, *cpe*, and *pfoA*), five hydrolytic enzyme-encoding genes (*nanH*, *nanI*, *nanJ*, *nagH*, and *colA*), the genes encoding four variants of the Cna collagen binding protein (*cnaA*, *cnaB*, *cnaC*, *cnaD*), three NE loci (NELoc-1–3), and the bacteriocin-encoding gene (perfrin) (see Supplementary Table S2). Genes with ≥90% nucleotide sequence identity and coverage compared to the NCBI-published ones were considered matches in this analysis (see Supplementary Table S2). Gene cluster visualizations for NEloc-1–3 were carried out using the R package geneviewer v. 0.1.8 “https://github.com/nvelden/geneviewer (accessed on 15 June 2024)”, and the associated heat maps were generated by “ComplexHeatmap” in the R package v. 2.15.4 [24].

### 2.5. Pangenome Analysis and Prediction of Prophages, Antibiotic Resistance Genes, and Plasmids

Pangenome analysis was performed using Roary v. 3.13.0 [25]. The aligned sequences of the core genome generated by Roary were used for phylogenetic analysis by FastTree v. 2.1.10 [26] and 1000 bootstrap replications. Visualization and annotation of the generated phylogenetic tree were implemented using ggtree v. 3.4.2 [27] and the R software v. 4.0.3 (R Core Team, 2020) [28]. Putative prophages were predicted using the PHASTEST web server v. 3.0 “https://phastest.ca/ (accessed on 21 June 2024)” [29], while plasmid prediction was conducted via PlasmidSeeker software v. 1.3.0 with default settings [30] and ABRicate v. 1.0.1 “https://github.com/tseemann/abricate (accessed on 12 July 2024)” with a best hit approach at nucleotide sequence identity ≥ 90% and query coverage threshold ≥ 70%. Finally, antibiotic resistance genes (ARGs) were identified using the read mapping-based tool SRST2 v. 0.2.0 [31], and the Comprehensive Antibiotic Resistance Database (CARD) v. 3.2.9 “https://card.mcmaster.ca/ (accessed on 20 July 2024)” [32].

## 3. Results

### 3.1. Sample Collection and Isolation of C. perfringens Strains

A total of 41 *C. perfringens* isolates including 26 from NE-affected and 15 from healthy broiler chicken flocks were subjected to WGS. Table 1 presents the name, year and origin of isolation, PFGE and type IV pilus profiles of isolates included in the current study. A total of 14 distinct PFGE profiles and 20 different type IV pilus profiles were identified, which represent the diversity among the *C. perfringens* strains analyzed in the present study.

### 3.2. Genome Assembly and Annotation

We first performed de novo assemblies of the genomes of the 41 *C. perfringens* isolates. Briefly, the average genome length, GC content, and number of coding sequences (CDSs) were 3,500,210 bp, 28%, and 3206, respectively. The average number of contigs per genome was 86, ranging from 29 to 209. Furthermore, the median N50 value was 200,369 bp, with the highest and lowest values being 721,013 bp and 48,863 bp, respectively. The median N50 value demonstrated a relatively contiguous short-read de novo assembly. Detailed statistics are presented in Table 1.

### 3.3. Pangenome Analysis and Genetic Relationships Between Isolates

**Figure 1:**
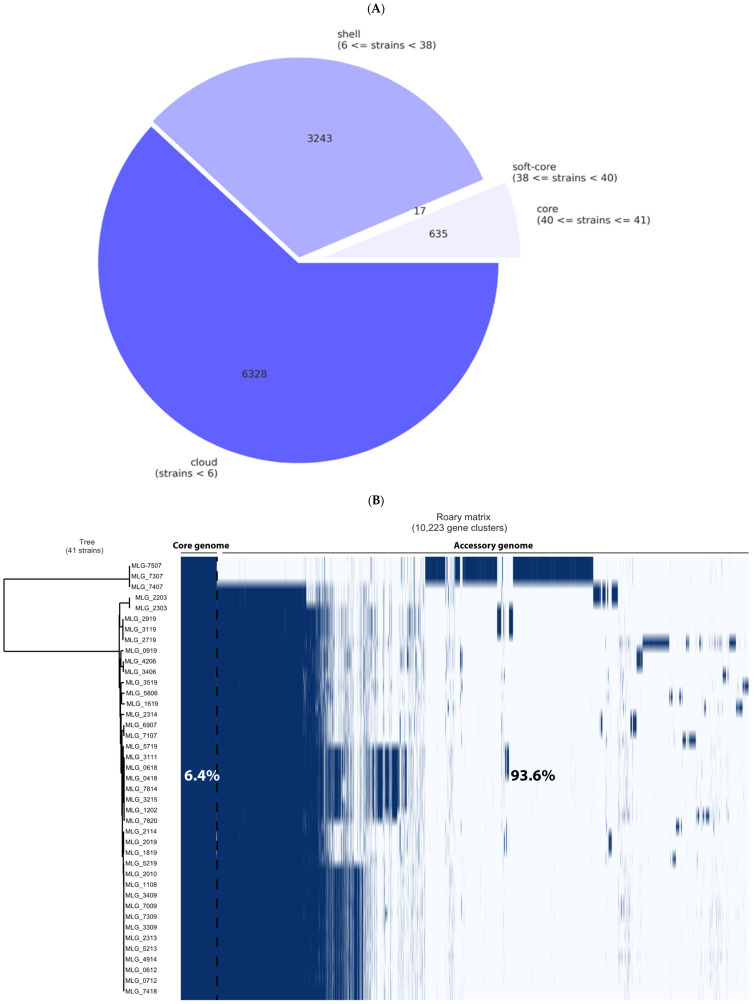
Results of the Roary pangenome analysis of 41 *C. perfringens* strains using a threshold of ≥95%. (**A**) Core, soft-core, shell, and cloud genes statistics. (**B**) Visualization of the pangenome analysis of 41 *C. perfringens* isolates along with the phylogenetic tree. The dark blue and light blue blocks represent the presence and absence of the genes, respectively.

Results generated from the Roary analysis showed that the pangenome of the *C. perfringens* strains consisted of 10,223 genes. Depending on their frequency among the sampled genomes, they were classified as core (present in 40–41 genomes), soft-core (present in 38–39 genomes), shell (present in six to 37 genomes), and cloud (present in less than six genomes) genes (Figure 1A). This classification resulted in 635 strict-core genes, 17 soft-core genes, 3243 shell genes, and 6328 cloud genes (Table 2). A total of 1917 unique genes (genes only present in one strain in this pangenome) were also found (see Supplementary Figure S1A–C). The low number of core genes (*n* = 635) present among 41 genomes indicated that our collection was highly diverse.

Regarding the phylogenetic tree structure, two distinct groups were identified. While the first group was composed of only three strains (MLG_7307, MLG_7407, and MLG_7507), all remaining isolates belonged to the latter group (Figure 1B). These three strains were collected during the same visit on one specific NE-affected farm, they do share an identical PFGE profile, while they differ regarding the type IV pilus profile. The second cluster consisted of 38 phylogenetically closely related isolates that were recovered from both healthy and NE-diseased frocks.

### 3.4. In Silico Profiling of Virulence Traits

**Figure 2:**
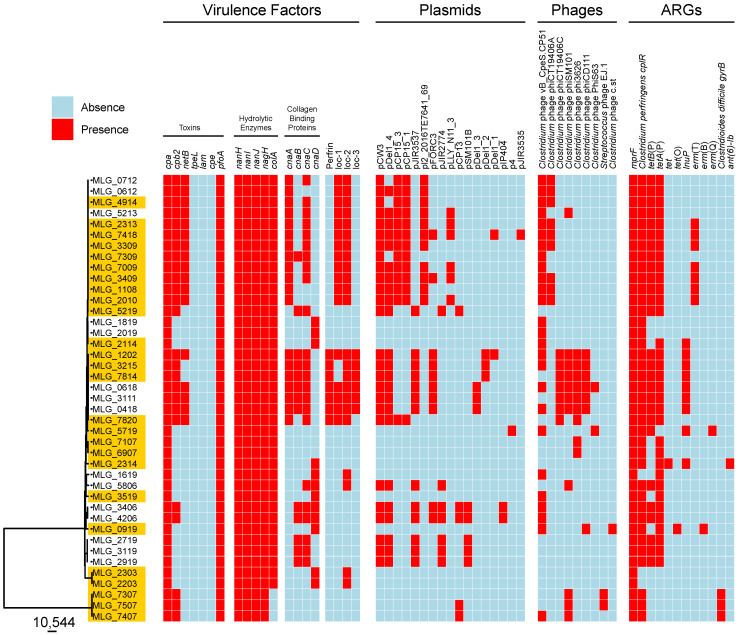
Heat map showing potential virulence factors, plasmids, antibiotic resistance genes, and bacteriophage profiles identified in 41 *C. perfringens* genomes. The presence of genes is indicated by cell colours: red (presence) and blue (absence). Orange-labelled strains were recovered from NE-affected flocks.

We next interrogated the genomes for the presence of 20 key *C. perfringens* virulence factors (see Supplementary Table S2). Results are presented in Figure 2 and Table 3. As expected, the genes encoding *C. perfringens* alpha-toxin (CPA) and perfringolysin O (PFO) were present in all strains (41/41, 100%). In contrast, toxin-encoding genes *tpeL*, *lam*, and *cpe* were not detected in any of the genomes screened, while *cpb2* and *netB* were found in 60% (25/41) and 41% (17/41) of strains, respectively. Of the genes encoding hydrolytic enzymes, three sialidase (NanH, NanI, and NanJ)- and a hyaluronidase (NagH)-encoding genes were found in all isolates, while the gene encoding the collagenase ColA was variable present (38/41, 92%). Differences were also observed for genes *cnaA*, *cnaB*, *cnaC*, and *cnaD* encoding putative collagen adhesin proteins, which were detected in 46%, 31%, 56%, and 29% of strains, respectively. Furthermore, seven strains (17%) possessed the bacteriocin perfrin-encoding gene. Among the three NE-associated loci (NELoc-1–3), NELoc-2 was the most frequently found (23/41, 56%), followed by NELoc-1 (17/41, 41%) and NELoc-3 (6/41, 14%). Globally, 51% of strains (21/41) harboured more than ten virulence factor-encoding genes. Of them, 62% (13/21) were isolated from diseased birds. As indicated in the previous section, two clusters were identified in the phylogenetic tree. The first cluster consisted of only three NE-diseased strains (MLG_7307, MLG_7407, and MLG_7507). Surprisingly, these isolates had fewer virulence factors, i.e., they possessed only seven virulence determinants out of the 20 investigated. Furthermore, they lacked NELoc-1–3, ColA, NetB, perfrin, and all variants of collagen adhesin protein-encoding genes.

### 3.5. Characterization of NELoc-1–3

**Figure 3:**
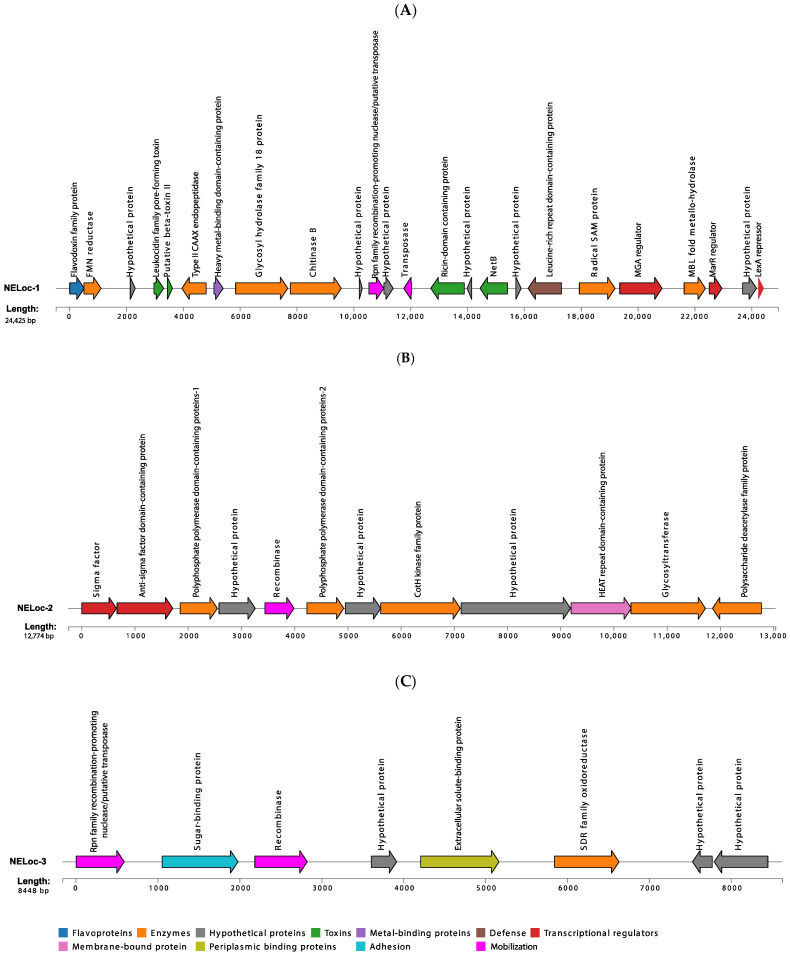
Visualization of the genetic composition of (**A**) NELoc-1, (**B**) NELoc-2, and (**C**) NELoc-3. Each arrow represents a predicted gene conserved in ≥70% of strains positive for the indicated locus. Predicted functional annotations and approximate size are shown above and below each gene, respectively. The arrows indicate the direction of transcription for each gene. Genes are colour-coded by their putative role based on sequence analyses.

A total of 41 different genes along with several hypothetical genes (with unknown biochemical functions) were identified in NELoc-1 (see Figure 3A and Supplementary Figure S2A). Among them were genes encoding for toxins (leukocidin family pore-forming toxin, putative beta-toxin II, NetB, and a ricin-domain containing protein), enzymes (e.g., FMN reductase, chitinase B, transposase, MBL fold metallo-hydrolase, and radical SAM protein), transcriptional regulators (e.g., LexA family transcriptional regulator and M protein trans-acting positive regulator (MGA)), one flavodoxin family protein, a heavy metal-binding domain-containing protein, and a leucine-rich repeat domain-containing protein (Figure 3A). Also, three pilus-associated structures (class C sortase, SpaH/EbpB family LPXTG-anchored major pilin, and SpaA isopeptide-forming pilin-related protein), two putative membrane proteins, and essential genes for protein biosynthesis such as elongation factor Tu and translation elongation factor EF-G were detected in several strains (see Supplementary Figure S2A). An interesting finding was the lack of *netB* in this locus in one isolate (MLG_7820).

Sequence analyses also revealed that NELoc-2 harboured ten different genes with known functions, including two variants of polyphosphate polymerase domain-containing proteins, one CotH kinase family protein, and a HEAT repeat domain-containing protein, which is a membrane-bound protein (see Figure 3B and Supplementary Figure S2B). Other genes included a sigma factor, an anti-sigma factor domain-containing protein, a recombinase family protein, and one glycosyltransferase, which were identified in 82% of strains (19/23) and a polysaccharide deacetylase family protein, which was predicted in 78% of isolates (18/23) positive for NELoc-2. The gene encoding the PBS lyase was identified in only one strain (MLG_0618) (see Supplementary Figure S2B).

Moreover, our results showed that NELoc-3 found in the strains analyzed was composed of eight genes, including one SDR family oxidoreductase, one extracellular solute-binding protein, a recombinase family protein, one sugar-binding protein, and an Rpn family recombination-promoting nuclease/putative transposase, all conserved across positive strains for this locus (6/6, 100%) (see Supplementary Figure S2C). Three hypothetical genes were also detected in NELoc-3 (Figure 3C).

The name of genes identified within each locus and their distribution across NE-diseased and healthy *C. perfringens* strains are presented in Figure S2A–C. The name, estimated size, and predicted function of the identified genes in the majority of isolates (≥70%) positive for the indicated locus are shown in Figure 3A–C.

### 3.6. ARG Prediction

Using read alignment to the CARD database; we identified 12 different ARGs across 41 *C. perfringens* genomes (Figure 2). Among them, the multiple peptide resistance factor (*mprF*) was the most prevalent (41/41, 100%), followed by *C. perfringens cplR* (38/41, 92%). Tetracycline resistance genes represented by *tet*, *tetA*(P), *tetB*(P), and *tet*(O) were also identified in 82% of strains (34/41), while the erythromycin resistance genes (*erm*(B), *erm*(Q), and *erm*(T)) were predicted in 22% of strains (9/41). Similarly, the *lnuP* gene, which encodes a putative lincosamide nucleotidyltransferase, was found in nine out of 41 strains (22%). Interestingly, *Clostridioides difficile gyrB* conferring resistance to fluoroquinolones was observed in three strains (MLG_7307, MLG_7407, and MLG_7507). In the updated version of the CARD database, sequences such as *gyrB* can potentially confer resistance based on point mutations within its sequence. Further phenotypic testing is needed. The gene encoding ANT(6)-Ib, an aminoglycoside nucleotidyltransferase, was identified in only one strain (MLG_2314).

Table 4 presents the name, description, and occurrence of the ARGs identified in the current study. The distribution of ARGs among the 41 *C. perfringens* genomes is shown in Figure 2.

### 3.7. Prophage Content of C. perfringens Isolates

Using the PHASTEST web server, we next identified nine different intact (score > 90) prophage regions within the *C. perfringens* genomes analyzed: *Clostridium* prophages vB_CpeS-CP51, phiCT19406A, phiCT19406C, phiSM101, phi3626, phiCD111, PhiS63, c-st, and *Streptococcus pneumoniae* prophage EJ-1, most of which encode endolysin and stage III sporulation protein D; thus, those prophages may play a role in *C. perfringens* sporulation and/or spore germination (Table 5). Among them, two prophages belonging to the *Siphoviridae* family were the most frequently found: *Clostridium* phage vB_CpeS-CP51 (also known as phiCP51) was found in 26 isolates (63%), while phiSM101 was found in 11 (26%). Other recurrent prophage-like sequences identified were phi3626 and phiCT19406A (9 out of 41 isolates; 22%), while phiCT19406C and phiCD111 were identified in seven isolates (17%). Interestingly, two strains (MLG_7307 and MLG_7507) possessed *Streptococcus* phage EJ-1 as well as several questionable prophage-like regions (scores 70–90) belonging to other bacterial genera, including *Bacillus* and *Lactobacillus*.

In summary, 83% of the isolates (34/41) contained one to six intact prophage regions. Table 5 and Figure 2 show the name, description, and distribution of the identified prophages across the 41 *C. perfringens* strains.

### 3.8. Presence of Plasmids in C. perfringens Isolates

We detected a total of 17 different plasmids in our isolate collection. Among them, pCW3-, pDel1_4-, pCP15_3-, pCP15_1-, and pJIR3537-like plasmids were the most frequently observed (63% to 31% of the isolates; Table 6 and Figure 2). Others included pl2_2016TE7641_69-, pFORC3-, pJIR2774-, pLLY_N11_3-, pCP13-, pSM101B-, pDel1_3-, pDel1_2-, pDel1_1-, pIP404, p4-, and pJIR3535-like plasmids (Table 6 and Figure 2). Most of these plasmids (10/17, 58%) harbour a *tcp* conjugation locus, which mediates horizontal gene transfer (HGT) between *C. perfringens* strains. Furthermore, several plasmids carried AGRs; notably plasmids pCW3, pDel1_4, and pJIR3537 possessed genes conferring resistance to tetracyclines. Regions encoding transposases, bacteriocins, and putative collagen adhesin proteins were found in some plasmids, such as pIP404 and pCP13. Similarly, pSM101B contained the gene encoding a BhlA/UviB family holin-like peptide; thus, potentially involved in endolysin and bacteriocin secretion, respectively.

In summary, 29 isolates (70%) possessed plasmids, and individual isolates had between one and nine (Figure 2). Of these 29 isolates, 17 were from NE-affected broiler chicken flocks (59%) and 12 strains were recovered from healthy birds (41%).

## 4. Discussion

Exploring the genome of *C. perfringens*, the main causative agent of NE in broiler chickens, is a crucial step in understanding the disease pathogenesis since it provides insights into the genetic factors driving virulence, resistance, and transmission, which can inform better prevention and control strategies [1,18]. Previous reports have emphasized the need to examine the *C. perfringens* genome to uncover potential virulence factors beyond the already identified toxins and other known elements contributing to its pathogenicity [1,18]. In this study, we investigated the genetic diversity of 41 *C. perfringens* strains of avian origin recovered in Quebec province, Canada, including 26 from NE-affected and 15 from healthy broiler chicken flocks. Using comparative genomics, we further investigated the presence of various virulence factors, ARGs, prophage regions, and plasmids, thus providing a comprehensive characterization of the genomic features of these isolates.

Our results suggest that the pangenome of our isolate collection remains open, supporting previous findings that it is challenging to define a *C. perfringens* core genome with high identity scores between strains [18,33]. A previous investigation by Camargo et al. [33] that evaluated 372 *C. perfringens* genomes from multiple locations and sources, including birds, using a threshold of ≥95%, identified only 959 core genome genes among 35,876 genes in pangenome (2.7%). Our analysis, using the same similarity threshold but a more restricted collection (only isolates from birds recovered in a single Canadian province), revealed a core genome composed of only 6.4% of the total pangenome genes. Thus, our results and previous ones [18,33] demonstrate that this pathogen has a relatively high level of genetic diversity, which aligns with its versatile lifestyle and ability to thrive in diverse hosts and environments, including the human and animal gut and soil. Since *C. perfringens* is distributed in various environments and is well known for the contribution of the mobilome to its virulence, its genetic composition can greatly vary depending on the host, environment, and bacterial interactions [34,35,36]. Furthermore, the high number of genes in the accessory genome highlights the need for future studies focused on this area to identify new virulence factors. While our current pangenome analysis has explored variable gene content, including mobile genetic elements such as phages and plasmids, much of the accessory genome remains unexamined in this investigation. Future investigations will be essential to fully understand *C. perfringens* genome diversity and its role in NE pathogenesis.

As previously indicated, three specific strains (MLG_7307, MLG_7407, and MLG_7507) formed a distinct cluster from the other isolates in the phylogenetic tree. Interestingly, they were recovered from the same NE-afflicted flock in Quebec; however, they harboured fewer virulence factors compared to the NE-diseased isolates belonging to the major clade. This suggests that these isolates may carry unknown virulence determinants, which play a pivotal role in NE pathogenesis as they might involve regulatory elements or reflect functional mechanisms not yet explored. Future work should focus on integrating transcriptomics, proteomics, or functional assays to identify these potential factors. As the avian host is also recognized to influence the occurrence and sometimes severity of the disease, considering host factors while conducting future studies would also be meaningful.

We examined in greater detail differences in virulence factor-encoding gene content in isolates of our collection. Our results showed that neither the isolates from NE-affected birds nor those recovered from healthy birds contained the TpeL-encoding gene, further supporting the idea that this toxin is not essential for NE pathogenesis [37]. Moreover, the gene *netB* was found with similar frequency in isolates from both healthy and diseased flocks (40% and 42%, respectively). This suggests that *netB*-positive strains from healthy flocks might exhibit virulence if predisposing factors, such as *Eimeria* infection, were present to trigger their expression. Therefore, it may be valuable to test the virulence potential of these 15 isolates under controlled experimental conditions. The absence of *netB* in 58% of isolates from NE-affected birds further supports the role of other virulence factors in disease pathogenesis. The *netB* gene is located in NELoc-1, which was first identified and characterized by Lepp et al., along with two other loci, NELoc-2 and NELoc-3 [14]. In addition to *netB*, the authors identified 36 extra genes in NELoc-1, including those predicted to encode two chitinases (chitinase A and B), two leukocidins, an internalin-like protein, and a ricin-domain protein [14]. Consistent with that description, we identified an MGA transcriptional regulator, MarR transcriptional regulator-encoding genes, radical SAM-domain protein, transposase, ricin-domain protein and two putative membrane proteins in the NELoc-1 of our isolates. However, we only detected one leukocidin family pore-forming toxin, while we did not find a gene encoding chitinase A in the NEloc-1 of our isolates.

Our findings revealed an anti-sigma factor domain-containing protein, two variants of polyphosphate polymerase domain-containing proteins, a PBS lyase, and a polysaccharide deacetylase family protein-encoding gene in NELoc-2. In addition, we identified a sigma factor, recombinase family protein, CotH kinase family protein, glycosyltransferase, and HEAT repeat domain-containing protein, consistent with earlier reports by Lepp et al. [14]. While these authors predicted the presence of genes encoding two putative VTC (vacuolar transporter chaperone)-domain superfamily proteins and two putative tubulin/FtsZ GTPases in NELoc-2 [14], these were not detected in our study.

When we evaluated NELoc-3, we identified one SDR family oxidoreductase, an Rpn (recombination-promoting nuclease/putative transposase) family, and a sugar-binding protein, in addition to the extracellular solute-binding protein and a recombinase family protein, which were previously identified by Lepp et al. [14]. Moreover, three extra genes, which were annotated as hypothetical, were found in our analysis (see Supplementary Figure S3).

The identification of NELoc-1–3 in isolates from healthy flocks raises intriguing questions about NE pathogenesis. One possibility is that these strains possessed the potential to cause disease but did not encounter the necessary predisposing factors, such as co-infections or immune suppression, to express their virulence. Another more likely hypothesis is that the presence of NELoc 1–3 alone is insufficient for *C. perfringens* to induce NE. It is plausible that additional factors, such as specific regulatory elements or virulence genes located on the chromosome or mobile genetic elements like plasmids, are required to fully activate the pathogenic mechanisms. These genes could interact with environmental or host-related triggers, and their absence may explain why these strains did not cause disease despite harbouring key loci. Further research into these complementary factors will be needed to identify the full genetic requirements for disease induction.

Resistance to antibiotics by *C. perfringens* has been documented over the years, and the bacterium is considered a multidrug-resistant pathogen [1]. However, the complete resistance profile of *C. perfringens* remains to be explored. So far, the most frequently reported antibiotic resistance in *C. perfringens* is tetracycline resistance, which is conferred by genes carried in several mobile genetic elements [34]. As shown here, tetracycline resistance genes were frequently observed in the genomes of the *C. perfringens* isolates evaluated here, with 82% of strains possessing genes *tet*, *tetA*(P), *tetB*(P), or *tet*(O). However, our results showed that gene *mprF* was the most common AGR, with all 41 *C. perfringens* isolates analyzed here possessing it. MprF is an integral membrane enzyme that neutralizes the membrane surface charge; thus, it drives resistance to cationic antimicrobial peptides and may play a key role in immune evasion systems [38]. Gene *mprF* was first identified in *Staphylococcus aureus* and has been implicated in the resistance of that pathogen to antibiotics such as vancomycin, gentamycin, and, more recently, daptomycin [39]. This AGR has also been reported in *C. perfringens*, and one study by Kiu et al. has shown that *mprF* was present in 100% of the isolates they characterized [18]. Genes *erm* B, Q, and T, associated with macrolide, lincosamide, and streptogramin (MLS) resistance [40], were found in our collection, although in fewer isolates (9/41; 22%). Interestingly, *erm*(T) is carried by plasmids in the *Streptococcus* genus [41]. However, previous studies proved that the *erm*(T)-carrying plasmid pRW35 of *S. pyogenes* has great potential for dissemination [42,43]. Therefore, the widespread distribution of *C. perfringens* in diverse niches would make this bacterium susceptible to various plasmid transfers. Finally, the *ant(6)-Ib* gene was found only in one strain (MLG_2314), which is similar to the findings of a previous study [18]. This gene was identified in *Campylobacter fetus* and named *ant(6)-Ib* based on its amino acid sequence homology to aminoglycoside nucleotidyltransferase ANT(6)-I [44]. The presence of this gene in the genome of *C. perfringens* further supports the carriage of a broad range of antibiotic resistance determinants by this bacterium, in addition to the variability in this carriage among strains of this bacterial species.

The role of mobile genetic elements such as prophages in *C. perfringens* is not fully understood. Here, we found that the most common prophage sequences in our isolates were previously described *C. perfringens* prophages phiCP51 (63%) and phiSM101 (26%) [45]. Commonly designated as vB_CpeS-CP51, phiCP51 was first identified in the *C. perfringens* strain 5147-97 and seems to be involved in sporulation due to possessing two open reading frames: stage III sporulation protein D and a putative sporulation sigma factor [46]. Phage phiSM101 has been identified in the genome of SM101, an enterotoxigenic type F strain of *C. perfringens* [47]. The presence of this prophage within our isolates might be an indication of HGT between different sub-populations of *C. perfringens* of poultry origin, especially considering that broiler chickens have recently been identified as an unsuspected reservoir of enterotoxigenic *C. perfringens* [5,48]. Overall, the majority (83%) of our isolates possessed prophage sequences. However, the number of prophage regions varied across strains, with occurrence rates ranging from 2% to 63%, further demonstrating the important level of genetic diversity that exists among the *C. perfringens* genomes and the potentially important role of HGT in the acquisition of new genes that could impact the pathogenicity of this bacterium [9].

## 5. Conclusions

The impact of NE caused by *C. perfringens* in the poultry industry has become increasingly significant, especially with the more restrictive use of antibiotics in poultry feed. Studying this causative agent will help identify other potential targets to prevent and treat the disease. In this study, we conducted genome analysis on a considerable, though still limited, number of *C. perfringens* isolates because of a small number of farms distributed in a restricted geographical area, to explore genomic diversity within this collection. However, we did not identify a specific genetic marker set that differentiates strains from healthy birds and those isolated from NE-diseased flocks, as key virulence loci were present in both groups. This suggests that factors beyond these loci, such as additional genetic elements, environmental conditions or concomitant health problems such as coccidiosis, may influence disease expression. While our findings offer valuable insights, further research involving a larger and more geographically diverse set of isolates, in addition to extending this comparison to the proteomes of virulent and avirulent strains to uncover novel pathogenicity-associated proteins is necessary to identify additional factors involved in NE pathogenesis.

## 6. Patents

Some genomic data presented in this article are the subject of a US Patent Application, 63/486,749, which was filed on 24 February 2023 and is titled “recombinant vaccine proteins for the prevention of avian necrotic enteritis”.

## Figures and Tables

**Table 1:** Strain profiles, genome assembly statistics, number of CDS, and BioProject_ID of 41 *C. perfringens* isolates included in this study.

Strain	Year of Isolation	Isolate Origin	Type IV Pilus Profile	PFGE Profile	Genome					No of CDSs	BioProject_ID
					Length (bp)	No of Contigs	Coverage (x)	GC (%)	N50		
MLG_7309	2010–2011	NE-affected	1	V1	3,603,633	204	85	28	51,385	3403	PRJNA1188553
MLG_7009	2010–2011	NE-affected	1	V2	3,577,078	100	93	28	197,277	3347	PRJNA1188553
MLG_1108	2010–2011	NE-affected	1	V3	3,546,273	101	88	28	190,425	3300	PRJNA1188553
MLG_3309	2010–2011	NE-affected	2	V3	3,538,464	144	77	28	58,373	3298	PRJNA1188553
MLG_3409	2010–2011	NE-affected	1	V3	3,551,654	100	87	28	189,683	3297	PRJNA1188553
MLG_2010	2010–2011	NE-affected	2	V3	3,551,275	104	92	28	152,768	3306	PRJNA1188553
MLG_2313	2010–2011	NE-affected	9	V3	3,545,125	88	84	28	177,971	3290	PRJNA734442
MLG_2114	2010–2011	NE-affected	4	V3	3,307,721	49	72	28.1	219,784	2940	PRJNA1188553
MLG_7418	2010–2011	NE-affected	2	V3	3,544,119	82	87	28	219,477	3286	PRJNA1188553
MLG_1202	2010–2011	NE-affected	8	V5	3,747,051	129	67	27.9	114,628	3572	PRJNA734442
MLG_3215	2010–2011	NE-affected	6	V5	3,680,156	96	83	28	170,870	3498	PRJNA1188553
MLG_7814	2010–2011	NE-affected	5	V6	3,687,085	124	60	28	80,413	3511	PRJNA734442
MLG_7820	2010–2011	NE-affected	5	V7	3,730,847	209	91	27.9	48,863	3570	PRJNA734442
MLG_2314	2010–2011	NE-affected	12	V10	3,291,552	43	75	28.1	291,371	2920	PRJNA1188553
MLG_4914	2010–2011	NE-affected	6	V12	3,579,960	152	77	28	82,578	3345	PRJNA1188553
MLG_5806	2010–2011	Healthy	6	C1	3,324,484	32	87	28.1	474,093	2963	PRJNA734442
MLG_4206	2010–2011	Healthy	2	C2	3,400,900	48	64	28	141,651	3040	PRJNA734442
MLG_3406	2010–2011	Healthy	7	C2	3,400,562	55	68	28	126,010	3039	PRJNA734442
MLG_5213	2010–2011	Healthy	6	C3	3,527,345	92	64	28	158,351	3269	PRJNA734442
MLG_0612	2010–2011	Healthy	6	C3	3,537,182	94	104	28	217,561	3283	PRJNA734442
MLG_0712	2010–2011	Healthy	6	C3	3,527,154	171	72	28	49,443	3287	PRJNA1188553
MLG_7307	2010–2011	NE-affected	13	V1	3,660,280	90	74	28.5	97,597	3296	PRJNA1188553
MLG_7407	2010–2011	NE-affected	14	V1	3,661,751	58	76	28.5	172,371	3293	PRJNA1188553
MLG_7507	2010–2011	NE-affected	15	V1	3,665,112	49	90	28.5	233,259	3292	PRJNA1188553
MLG_6907	2010–2011	NE-affected	1	V2	3,281,089	30	97	28.1	699,417	2921	PRJNA1188553
MLG_7107	2010–2011	NE-affected	1	V2	3,281,533	36	89	28.1	222,145	2920	PRJNA1188553
MLG_2203	2010–2011	NE-affected	16	V3	3,281,091	36	83	28.1	219,382	2880	PRJNA1188553
MLG_2303	2010–2011	NE-affected	17	V3	3,283,967	29	66	28.1	721,013	2881	PRJNA1188553
MLG_3519	2010–2011	NE-affected	4	V5	3,227,573	33	64	28.2	255,420	2867	PRJNA1188553
MLG_0919	2010–2011	NE-affected	18	V6	3,892,868	148	77	28.5	62,235	3661	PRJNA1188553
MLG_5719	2010–2011	NE-affected	1	V7	3,379,598	58	70	28.2	133,605	3023	PRJNA1188553
MLG_5219	2010–2011	NE-affected	19	V8	3,291,656	99	81	28.1	62,796	2953	PRJNA1188553
MLG_3119	2010–2011	Healthy	7	C1	3,435,369	42	86	28	265,064	3053	PRJNA734442
MLG_2919	2010–2011	Healthy	20	C1	3,434,769	42	113	28	397,745	3051	PRJNA734442
MLG_2719	2010–2011	Healthy	21	C1	3,434,532	39	108	28	413,284	3049	PRJNA734442
MLG_2019	2010–2011	Healthy	3	C2	3,233,362	60	82	28.2	127,551	2880	PRJNA734442
MLG_1819	2010–2011	Healthy	3	C3	3,234,658	48	65	28.2	198,347	2884	PRJNA1188553
MLG_1619	2010–2011	Healthy	22	C3	3,269,123	39	94	28.1	232,490	2913	PRJNA734442
MLG_0418	2010–2011	Healthy	5	C4	3,788,295	115	94	28	114,406	3622	PRJNA1188553
MLG_0618	2010–2011	Healthy	8	C4	3,782,263	143	126	28	71,277	3624	PRJNA1188553
MLG_3111	2010–2011	Healthy	5	C5	3,790,123	124	70	28	102,785	3633	PRJNA1188553
AVERAGE					3,500,210	86	83	28	200,369	3206	

**Table 2:** Genome diversity among the 41 *C. perfringens* strains analyzed.

Description	Description	Nb of Strains	Nb of Genes	Pangenome (%)
Core genome	Core genes	(99% ≤ strains ≤ 100%)	635	(*n* = 652), 6.4%
	Soft-core genes	(95% ≤ strains < 99%)	17	
Accessory genome	Shell genes	(15% ≤ strains < 95%)	3243	(*n* = 9571), 93.6%
	Cloud genes	(0% ≤ strains < 15%)	6328	
Pangenome	Pangenome	(0% ≤ strains ≤ 100%)	10,223	100%

**Table 3:** Name and occurrence of putative NE-associated virulence factors among *C. perfringens* strains isolated from either healthy or NE-afflicted birds.

	Toxin	Enzyme	Enzyme	Enzyme	Enzyme	Toxin	Enzyme	Collagen Binding Protein	Collagen Binding Protein	Collagen Binding Protein	Collagen Binding Protein	Bacteriocin	Toxin	Toxin	NE Loci	NE Loci	NE Loci	Toxin	Toxin	Toxin
Isolate origin	*cpa*	*nanH*	*nanI*	*nanJ*	*nagH*	*pfoA*	*colA*	*cnaA*	*cnaB*	*cnaC*	*cnaD*	*perfrin*	*netB*	*cpb2*	NE Loc-1	NE Loc-2	NE Loc-3	*tpeL*	*cpe*	*lam*
NE-affected (*n* = 26)	100%	100%	100%	100%	100%	100%	88%	50%	19%	46%	23%	15%	42%	65%	42%	57%	11%	0%	0%	0%
Healthy (*n* = 15)	100%	100%	100%	100%	100%	100%	100%	40%	53%	73%	40%	20%	40%	53%	40%	53%	20%	0%	0%	0%
Total (*n* = 41)	100%	100%	100%	100%	100%	100%	92%	46%	31%	56%	29%	17%	41%	60%	41%	56%	14%	0%	0%	0%

The green, blue, and orange background colours represent the absolute presence, variable presence, and absence, respectively, of the indicated genes among the 41 *C. perfringens* strains analyzed.

**Table 4:** Name, description, and occurrence of ARGs predicted in the present study. The total percentage of occurrence was calculated based on the presence of the indicated antibiotic resistance determinant in all strains.

ARGs	Description	Occurrence		
		NE-Affected Isolates	Healthy Isolates	Total
*mprF*	Resistance to cationic peptides	100%	100%	100%
*C. perfringens cplR*	Resistance to pleuromutilin, lincosamide, and streptogramin A	92%	93%	92%
*tetA*(P)	Resistance to tetracycline antibiotic	76%	86%	80%
*tetB*(P)	Resistance to tetracycline antibiotic	57%	80%	65%
*tet*	Resistance to tetracycline antibiotic	3%	0%	2%
*tet*(O)	Resistance to tetracycline antibiotic	3%	0%	2%
*erm*(B)	Resistance to macrolide, lincosamide, and streptogramin	3%	0%	2%
*erm*(Q)	Resistance to macrolide, lincosamide, and streptogramin	3%	0%	2%
*erm*(T)	Resistance to macrolide, lincosamide, and streptogramin	27%	0%	17%
*lnuP*	Lincosamide nucleotidyltransferase (LNU)	23%	20%	22%
*C. difficile gyrB*	Resistance to fluoroquinolones	11%	0%	7%
*ant(6)-Ib*	Aminoglycoside nucleotidyltransferase	3%	0%	2%

**Table 5:** Name, description, and occurrence of the prophage regions predicted in the present study. The total percentage of occurrence was computed as the total number of strains positive for the aforementioned prophage region.

Prophage	Description	Occurrence		
		NE-Affected Isolates	Healthy Isolates	Total
phiCP51	Holin, amidase, endolysin, stage III sporulation protein D, and putative sporulation sigma factor production	61%	66%	63%
phiCT19406A	Endolysin production	27%	13%	22%
phiCT19406C	Endolysin production	15%	20%	17%
phiSM101	Endolysin production	23%	33%	26%
phi3626	Endolysin production, stage III sporulation protein D	23%	20%	22%
phiCD111	Endolysin production	15%	20%	17%
PhiS63	Endolysin; glycosyl-hydrolase production	3%	6%	4%
*Streptococcus* phage EJ-1	Endolysin production	7%	0%	4%
*Clostridium* phage c-st	Endolysin production	3%	0%	2%

**Table 6:** Name, size, description, and frequency of *C. perfringens* plasmids among the strains analyzed. The total percentage of occurrence was calculated based on the existence of the mentioned plasmid in both healthy and diseased strains.

Name and NCBI Reference Sequence	Size (bp)	Description	Occurrence		
			NE-Affected Isolates	Healthy Isolates	Total
pCW3-like plasmid (NC_010937.1)	47,263	*tcp* conjugation locus Tetracycline-resistance determinant Putative transposase	53%	80%	63%
pDel1_4-like plasmid (NZ_CP019580.1)	49,728	*tcp* conjugation locus Tetracycline-resistance determinant Putative transposase	46%	73%	56%
pCP15_3-like plasmid (NZ_CP019570.1)	3843	-	38%	20%	31%
pCP15_1-like plasmid (NZ_CP019568.1)	3202	-	38%	20%	31%
pJIR3537-like plasmid (NZ_CP025504.1)	48,779	Tetracycline-resistance determinant *tcp* conjugation locus Putative transposase	15%	60%	31%
pl2_2016TE7641_69-like plasmid (NZ_MN503253.1)	82,669	*tcp* conjugation locus Tn3 family transposase Collagen binding domain-containing protein	30%	20%	26%
pFORC3-like plasmid (NZ_CP009558.1)	56,577	*tcp* conjugation locus Tetracycline-resistance determinant Putative transposase	19%	33%	24%
pJIR2774-like plasmid (DQ338473.1)	14,640	*tcp* conjugation locus	3%	40%	17%
pLLY_N11_3-like plasmid (NZ_CP023413.1)	72,060	*tcp* conjugation locus Tetracycline-resistance determinant Putative transposase	19%	6%	14%
pCP13-like plasmid (AP003515.1)	54,310	Probable collagen adhesin Probable transposase Resolvase	11%	13%	12%
pSM101B-like plasmid (NC_008264.1)	12,206	BhlA/UviB family holin-like peptide	0%	33%	12%
pDel1_3-like plasmid (NZ_CP019579.1)	49,582	Holin family protein IS630 transposase-related protein Chloramphenicol resistance protein	0%	20%	7%
pDel1_2-like plasmid (NZ_CP019578.1)	69,827	*tcp* conjugation locus Putative transposase	11%	0%	7%
pDel1_1-like plasmid (NZ_CP019577.1)	82,596	*tcp* conjugation locus Putative transposase Collagen binding domain-containing protein	7%	0%	4%
pIP404-like plasmid (NC_001388.1)	10,206	Bacteriocin UviB	0%	13%	4%
p4-like plasmid (NZ_MK275622.1)	40,125	IS1595 family transposase	3%	0%	2%
pJIR3535-like plasmid (NZ_CP025502.1)	81,826	*tcp* conjugation locus Tn3 family transposase Collagen binding domain-containing protein	3%	0%	2%

## Data Availability

Data are available in the NCBI database under the BioProject accession numbers PRJNA734442 and PRJNA1188553.

## Supplementary Materials

The following supporting information can be downloaded at: https://www.mdpi.com/article/10.3390/microorganisms12122624/s1, Table S1: Name and NCBI reference sequence of *C. perfringens* strains used for genome annotation; Table S2: Name and GenBank accession number of *C. perfringens* virulence factors documented in the present study; Figure S1: Results of the Roary pangenome analysis of 41 *C. perfringens* strains using a minimum protein percentage identity threshold of ≥95%; Figure S2: Heat maps presenting the identified genes within (A) NELoc-1, (B) NELoc-2, and (C) NELoc-3 and their distribution among *C. perfringens* strains using “ComplexHeatmap” in the R package; Figure S3: Comparison between (A) the previous findings by Lepp et al. [14] and (B) our results regarding NEloc-3.

## References

[1] Kiu R., Hall L.J. (2018). An update on the human and animal enteric pathogen *Clostridium perfringens*. Emerg. Microbes Infect..

[2] Mehdizadeh Gohari I., Navarro M.A., Li J., Shrestha A., Uzal F., McClane B.A. (2021). Pathogenicity and virulence of *Clostridium perfringens*. Virulence.

[3] Fathima S., Hakeem W.G.A., Shanmugasundaram R., Selvaraj R.K. (2022). Necrotic Enteritis in Broiler Chickens: A Review on the Pathogen, Pathogenesis, and Prevention. Microorganisms.

[4] Alizadeh M., Shojadoost B., Boodhoo N., Astill J., Taha-Abdelaziz K., Hodgins D.C., Kulkarni R.R., Sharif S. (2021). Necrotic enteritis in chickens: A review of pathogenesis, immune responses and prevention, focusing on probiotics and vaccination. Anim. Health Res. Rev..

[5] Xu W., Wang H., Liu L., Miao Z., Huo Y., Zhong Z. (2021). Prevalence and characterization of *Clostridium perfringens* isolated from different chicken farms in China. Anaerobe.

[6] Mwangi S., Timmons J., Fitz-Coy S., Parveen S. (2019). Characterization of *Clostridium perfringens* recovered from broiler chicken affected by necrotic enteritis. Poult. Sci..

[7] Chalmers G., Martin S.W., Prescott J.F., Boerlin P. (2008). Typing of *Clostridium perfringens* by multiple-locus variable number of tandem repeats analysis. Vet. Microbiol..

[8] De Cesare A., Borilova G., Svobodova I., Bondioli V., Manfreda G. (2009). *Clostridium perfringens* occurrence and ribotypes in healthy broilers reared in different European countries. Poult. Sci..

[9] Lacey J.A., Allnutt T.R., Vezina B., Van T.T.H., Stent T., Han X., Rood J.I., Wade B., Keyburn A.L., Seemann T. (2018). Whole genome analysis reveals the diversity and evolutionary relationships between necrotic enteritis-causing strains of *Clostridium perfringens*. BMC Genom..

[10] Lepp D., Gong J., Songer J.G., Boerlin P., Parreira V.R., Prescott J.F. (2013). Identification of accessory genome regions in poultry *Clostridium perfringens* isolates carrying the *netB* plasmid. J. Bacteriol..

[11] Gaucher M.L., Perron G.G., Arsenault J., Letellier A., Boulianne M., Quessy S. (2017). Recurring Necrotic Enteritis Outbreaks in Commercial Broiler Chicken Flocks Strongly Influence Toxin Gene Carriage and Species Richness in the Resident *Clostridium perfringens* Population. Front. Microbiol..

[12] Park M., Deck J., Foley S.L., Nayak R., Songer J.G., Seibel J.R., Khan S.A., Rooney A.P., Hecht D.W., Rafii F. (2016). Diversity of *Clostridium perfringens* isolates from various sources and prevalence of conjugative plasmids. Anaerobe.

[13] Parreira V.R., Costa M., Eikmeyer F., Blom J., Prescott J.F. (2012). Sequence of two plasmids from *Clostridium perfringens* chicken necrotic enteritis isolates and comparison with *C. perfringens* conjugative plasmids. PLoS ONE.

[14] Lepp D., Roxas B., Parreira V.R., Marri P.R., Rosey E.L., Gong J., Songer J.G., Vedantam G., Prescott J.F. (2010). Identification of novel pathogenicity loci in *Clostridium perfringens* strains that cause avian necrotic enteritis. PLoS ONE.

[15] Zhou H., Lepp D., Pei Y., Liu M., Yin X., Ma R., Prescott J.F., Gong J. (2017). Influence of pCP1NetB ancillary genes on the virulence of *Clostridium perfringens* poultry necrotic enteritis strain CP1. Gut Pathog..

[16] Barbara A.J., Trinh H.T., Glock R.D., Glenn Songer J. (2008). Necrotic enteritis-producing strains of *Clostridium perfringens* displace non-necrotic enteritis strains from the gut of chicks. Vet. Microbiol..

[17] Myers G.S., Rasko D.A., Cheung J.K., Ravel J., Seshadri R., DeBoy R.T., Ren Q., Varga J., Awad M.M., Brinkac L.M. (2006). Skewed genomic variability in strains of the toxigenic bacterial pathogen, *Clostridium perfringens*. Genome Res..

[18] Kiu R., Caim S., Alexander S., Pachori P., Hall L.J. (2017). Probing Genomic Aspects of the Multi-Host Pathogen *Clostridium perfringens* Reveals Significant Pangenome Diversity, and a Diverse Array of Virulence Factors. Front. Microbiol..

[19] Keyburn A.L., Sheedy S.A., Ford M.E., Williamson M.M., Awad M.M., Rood J.I., Moore R.J. (2006). Alpha-toxin of *Clostridium perfringens* is not an essential virulence factor in necrotic enteritis in chickens. Infect. Immun..

[20] Meniaï I., Thibodeau A., Quessy S., Parreira V.R., Fravalo P., Beauchamp G., Gaucher M.L. (2021). Putative antigenic proteins identified by comparative and subtractive reverse vaccinology in necrotic enteritis-causing *Clostridium perfringens* isolated from broiler chickens. BMC Genom..

[21] Coil D., Jospin G., Darling A.E. (2015). A5-miseq: An updated pipeline to assemble microbial genomes from Illumina MiSeq data. Bioinformatics.

[22] Seemann T. (2014). Prokka: Rapid prokaryotic genome annotation. Bioinformatics.

[23] Altschul S.F., Gish W., Miller W., Myers E.W., Lipman D.J. (1990). Basic local alignment search tool. J. Mol. Biol..

[24] Gu Z., Eils R., Schlesner M. (2016). Complex heatmaps reveal patterns and correlations in multidimensional genomic data. Bioinformatics.

[25] Page A.J., Cummins C.A., Hunt M., Wong V.K., Reuter S., Holden M.T., Fookes M., Falush D., Keane J.A., Parkhill J. (2015). Roary: Rapid large-scale prokaryote pan genome analysis. Bioinformatics.

[26] Price M.N., Dehal P.S., Arkin A.P. (2010). FastTree 2–approximately maximum-likelihood trees for large alignments. PLoS ONE.

[27] Xu S., Li L., Luo X., Chen M., Tang W., Zhan L., Dai Z., Lam T.T., Guan Y., Yu G. (2022). Ggtree: A serialized data object for visualization of a phylogenetic tree and annotation data. Imeta.

[28] R Core Team R: A Language and Environment for Statistical Computing. (R Foundation for Statistical Computing, 2013). https://www.r-project.org/.

[29] Wishart D.S., Han S., Saha S., Oler E., Peters H., Grant J.R., Stothard P., Gautam V. (2023). PHASTEST: Faster than PHASTER, better than PHAST. Nucleic Acids Res..

[30] Roosaare M., Puustusmaa M., Möls M., Vaher M., Remm M. (2018). PlasmidSeeker: Identification of known plasmids from bacterial whole genome sequencing reads. PeerJ.

[31] Inouye M., Dashnow H., Raven L.A., Schultz M.B., Pope B.J., Tomita T., Zobel J., Holt K.E. (2014). SRST2: Rapid genomic surveillance for public health and hospital microbiology labs. Genome Med..

[32] Jia B., Raphenya A.R., Alcock B., Waglechner N., Guo P., Tsang K.K., Lago B.A., Dave B.M., Pereira S., Sharma A.N. (2017). CARD 2017: Expansion and model-centric curation of the comprehensive antibiotic resistance database. Nucleic Acids Res..

[33] Camargo A., Guerrero-Araya E., Castañeda S., Vega L., Cardenas-Alvarez M.X., Rodríguez C., Paredes-Sabja D., Ramírez J.D., Muñoz M. (2022). Intra-species diversity of *Clostridium perfringens*: A diverse genetic repertoire reveals its pathogenic potential. Front. Microbiol..

[34] Adams V., Han X., Lyras D., Rood J.I. (2018). Antibiotic resistance plasmids and mobile genetic elements of *Clostridium perfringens*. Plasmid.

[35] Lacey J.A., Johanesen P.A., Lyras D., Moore R.J. (2019). In silico Identification of Novel Toxin Homologs and Associated Mobile Genetic Elements in *Clostridium perfringens*. Pathogens.

[36] Wisniewski J.A., Rood J.I. (2017). The Tcp conjugation system of *Clostridium perfringens*. Plasmid.

[37] Yang W.Y., Chou C.H., Wang C. (2018). Characterization of toxin genes and quantitative analysis of *netB* in necrotic enteritis (NE)-producing and non-NE-producing *Clostridium perfringens* isolated from chickens. Anaerobe.

[38] Yin J., Meng Q., Cheng D., Fu J., Luo Q., Liu Y., Yu Z. (2020). Mechanisms of bactericidal action and resistance of polymyxins for Gram-positive bacteria. Appl. Microbiol. Biotechnol..

[39] Ernst C.M., Peschel A. (2011). Broad-spectrum antimicrobial peptide resistance by MprF-mediated aminoacylation and flipping of phospholipids. Mol. Microbiol..

[40] Feßler A.T., Wang Y., Wu C., Schwarz S. (2018). Mobile macrolide resistance genes in staphylococci. Plasmid.

[41] Yu R., Xu Y., Schwarz S., Shang Y., Yuan X., Zhang Y., Li D., Du X.D. (2022). *erm*(T)-Mediated Macrolide-Lincosamide Resistance in *Streptococcus suis*. Microbiol. Spectr..

[42] Woodbury R.L., Klammer K.A., Xiong Y., Bailiff T., Glennen A., Bartkus J.M., Lynfield R., Van Beneden C., Beall B.W. (2008). Plasmid-Borne *erm*(T) from invasive, macrolide-resistant *Streptococcus pyogenes* strains. Antimicrob. Agents Chemother..

[43] Varaldo P.E., Montanari M.P., Giovanetti E. (2009). Genetic elements responsible for erythromycin resistance in streptococci. Antimicrob. Agents Chemother..

[44] Abril C., Brodard I., Perreten V. (2010). Two novel antibiotic resistance genes, *tet*(44) and *ant*(6)-Ib, are located within a transferable pathogenicity island in *Campylobacter fetus* subsp. *fetus*. Antimicrob. Agents Chemother..

[45] Feng Y., Fan X., Zhu L., Yang X., Liu Y., Gao S., Jin X., Liu D., Ding J., Guo Y. (2020). Phylogenetic and genomic analysis reveals high genomic openness and genetic diversity of *Clostridium perfringens*. Microb. Genom..

[46] Gervasi T., Curto R.L., Narbad A., Mayer M.J. (2013). Complete genome sequence of ΦCP51, a temperate bacteriophage of *Clostridium perfringens*. Arch. Virol..

[47] Nariya H., Miyata S., Tamai E., Sekiya H., Maki J., Okabe A. (2011). Identification and characterization of a putative endolysin encoded by episomal phage phiSM101 of *Clostridium perfringens*. Appl. Microbiol. Biotechnol..

[48] Afshari A., Jamshidi A., Razmyar J., Rad M. (2015). Genotyping of *Clostridium perfringens* isolated from broiler meat in northeastern of Iran. Vet. Res. Forum.

