# Supplementary material for: Genomic Diversity and Virulence Factors of Clostridium perfringens Isolated from Healthy and Necrotic Enteritis-Affected Broiler Chicken Farms in Quebec Province
Source: Microorganisms. 2024 Dec 18;12(12):2624. doi: 10.3390/microorganisms12122624 (PMC11677781; doi:10.3390/microorganisms12122624)
Supplement: Supplementary file 1 [file microorganisms-12-02624-s001.zip › microorganisms-3366052-supplement.pdf]

Table S1. Name and NCBI reference sequence of *C. perfringens* strains used for genome annotation.

Name	NCBI reference sequence
Clostridium perfringens strain LLY_N11 chromosome	NZ_CP023410.1
Clostridium perfringens isolate MGYG-HGUT-02372 chromosome 1	NZ_LR698985.1
Clostridium perfringens strain JXJA17 isolate Intestine chromosome	NZ_CP028149.1
Clostridium perfringens strain EHE-NE18 chromosome	NZ_CP025501.1
Clostridium perfringens strain Del1 chromosome	NZ_CP019576.1
Clostridium perfringens strain FORC_025 chromosome	NZ_CP013101.1
Clostridium perfringens strain JP838 chromosome	NZ_CP010994.1
Clostridium perfringens strain JP55 chromosome	NZ_CP010993.1
Clostridium perfringens strain CBA7123 chromosome	NZ_AP017630.1
Clostridium perfringens SM101	NC_008262.1
Clostridium perfringens NCTC 8239 isolate	NZ_CABPRS010000001.1
Clostridium perfringens ATCC 13124	NC_008261.1
Clostridium perfringens CP4	NZ_LIYI00000000.1
Clostridium perfringens strain CPI 18-6 chromosome	CP075979.1
Clostridium perfringens strain Cp1 chromosome	CP120689.1
Clostridium perfringens strain CP15	NZ_CP019468.1
Clostridium perfringens str. 13	NC_003366.1

Table S2. Name and GenBank accession number of *C. perfringens* virulence factors documented in the present study. *GenBank accession numbers were associated with the amino acid sequence of the indicated virulence factor.

**Name**	**GenBank accession number**
cpa	X17300.1
cpb2	EGT3607767.1*
netB	KY923245.1
tpeL	AB262081.1
lam	D45904.1
cpe	M98037.1
pfoA	EF165974.1
nanH	UBK86913.1*
nanI	UBK86719.1*
nanJ	OQ095367.1
nagH	M81878.1
colA	MW393544.1
cnaA	KT749987.1
cnaB	BAB62495.1*
cnaC	ABC96287.1*
cnaD	ABG83269.1*
Bacteriocin perfrin	HQ666823.1
NELoc-1	JF837812.1
NELoc-2	JF837813.1
NELoc-3	JF837814.1

(A)

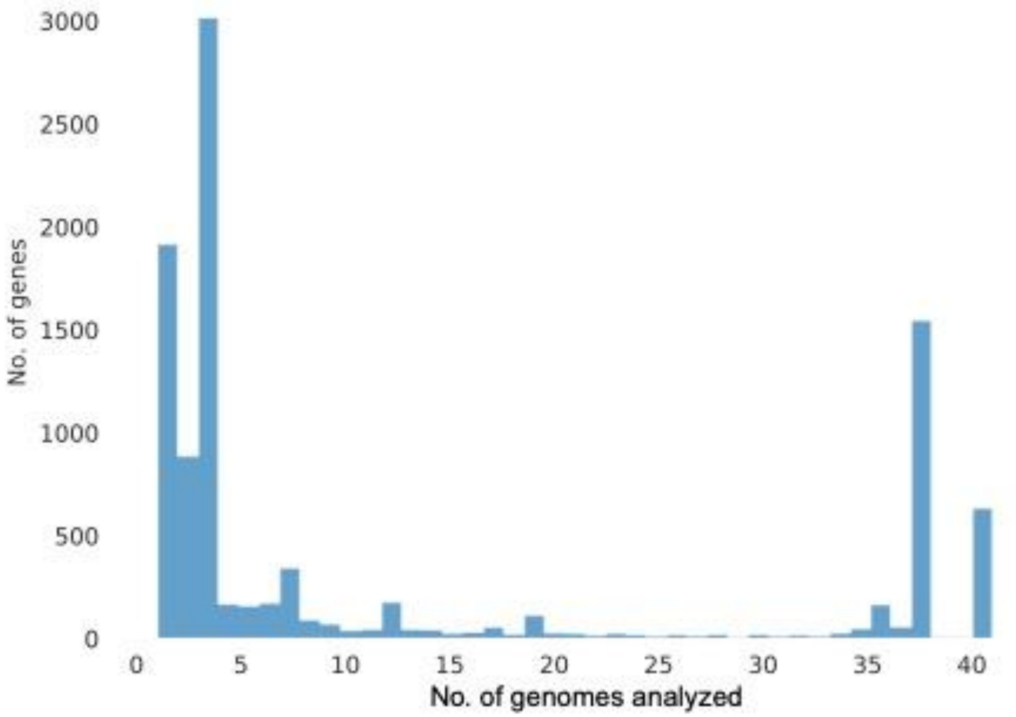

No. of genes
No. of genomes analyzed

(B)

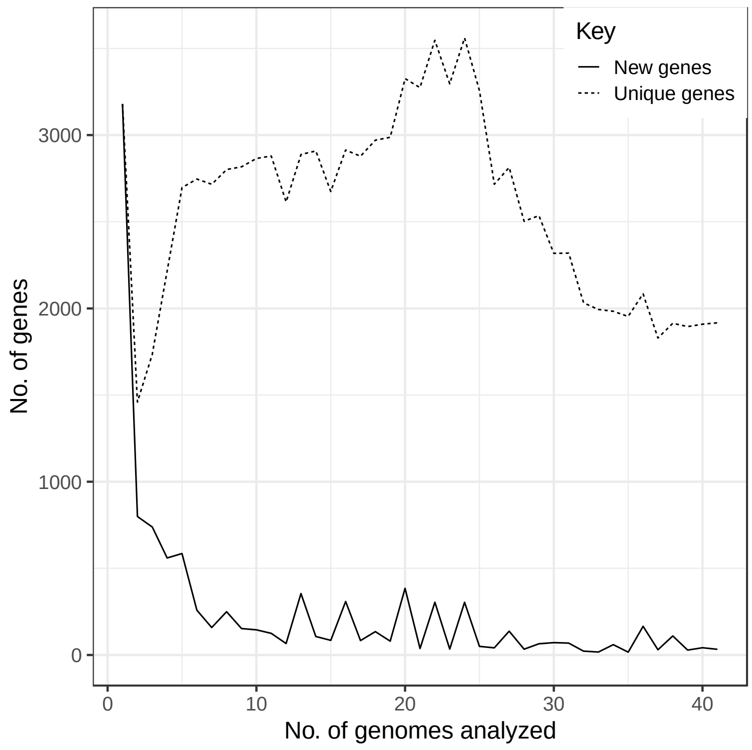

Key
New genes
Unique genes
No. of genes
No. of genomes analyzed

(C)

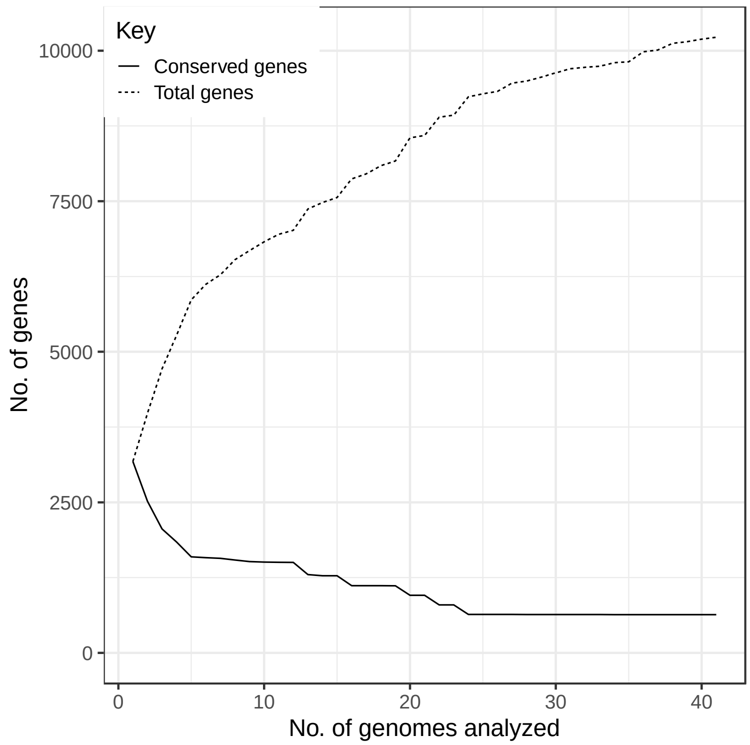

Figure S1. Results of the Roary pangenome analysis of 41 *C. perfringens* strains using a minimum protein percentage identity threshold of ≥95%. (A) Frequency bar graphs of the number of genes against the number of genomes. (B) Number of new genes and unique genes along pangenome calculation. (C) Number of conserved genes and total genes along pangenome computation.

(A)

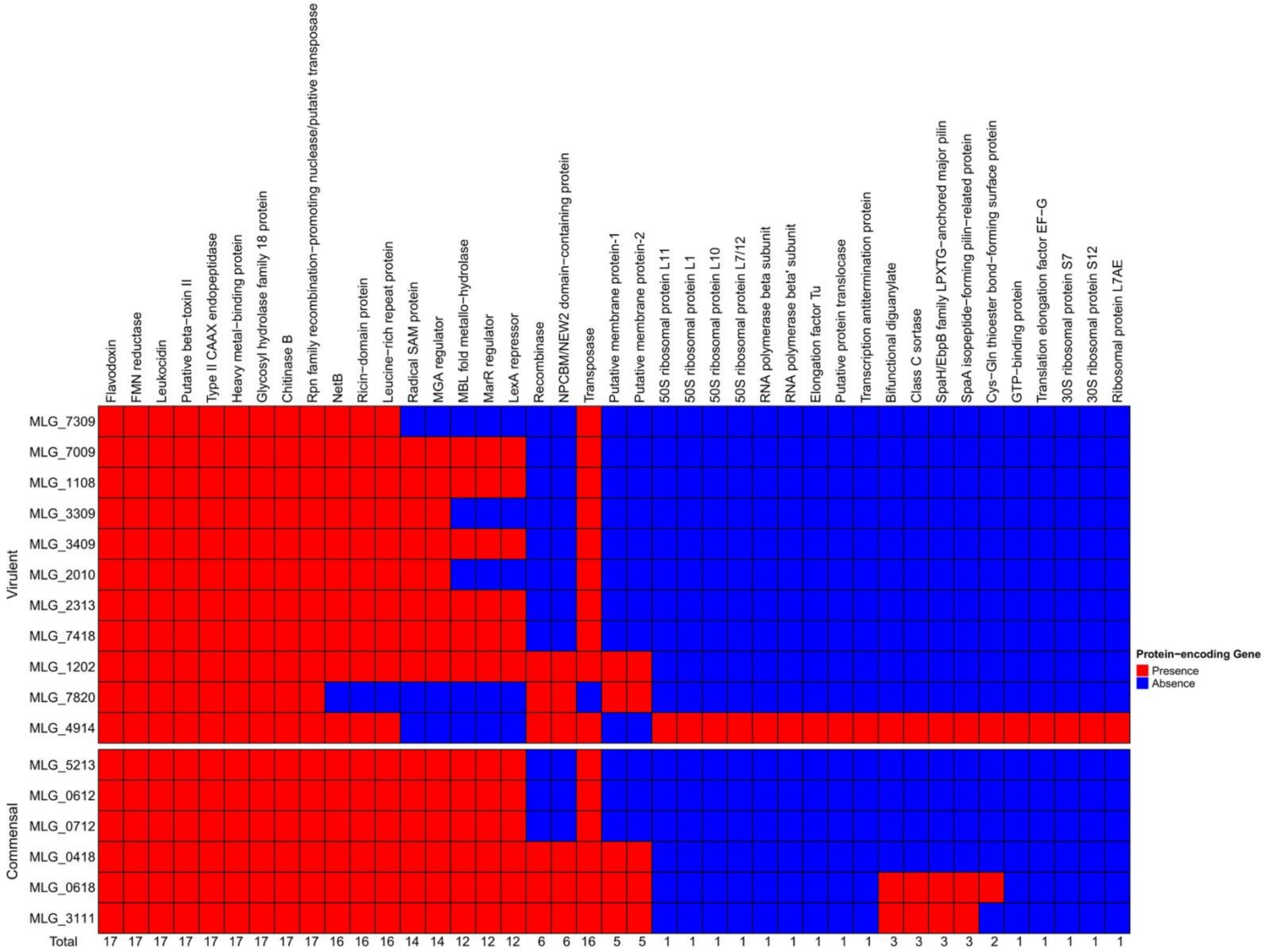
Flavodoxin
FMN reductase
Leukocidin
Putative beta−toxin II
Type II CAAX endopeptidase
Heavy metal−binding protein
Glycosyl hydrolase family 18 protein
Chitinase B
Rpn family recombination−promoting nuclease/putative transposase
NetB
Ricin−domain protein
Leucine−rich repeat protein
Radical SAM protein
MGA regulator
MBL fold metallo−hydrolase
MarR regulator
LexA repressor
Recombinase
NPCBM/NEW2 domain−containing protein
Transposase
Putative membrane protein-1
Putative membrane protein-2
50S ribosomal protein L11
50S ribosomal protein L1
50S ribosomal protein L10
50S ribosomal protein L7/12
RNA polymerase beta subunit
RNA polymerase beta' subunit
Elongation factor Tu
Putative protein translocase
Transcription antitermination protein
Bifunctional diguanylate
Class C sortase
SpaH/EbpB family LPXTG−anchored major pilin
SpaA isopeptide−forming pilin−related protein
Cys−Gln thioester bond−forming surface protein
GTP−binding protein
Translation elongation factor EF−G
30S ribosomal protein S7
30S ribosomal protein S12
Ribosomal protein L7AE
Virulent
MLG_7309
MLG_7009
MLG_1108
MLG_3309
MLG_3409
MLG_2010
MLG_2313
MLG_7418
MLG_1202
MLG_7820
MLG_4914
Commensal
MLG_5213
MLG_0612
MLG_0712
MLG_0418
MLG_0618
MLG_3111
Total
17 17 17 17 17 17 17 17 17 16 16 16 14 14 12 12 12 6 6 16 5 5 1 1 1 1 1 1 1 1 1 3 3 3 3 2 1 1 1 1 1
Protein−encoding Gene
Presence
Absence

(B)

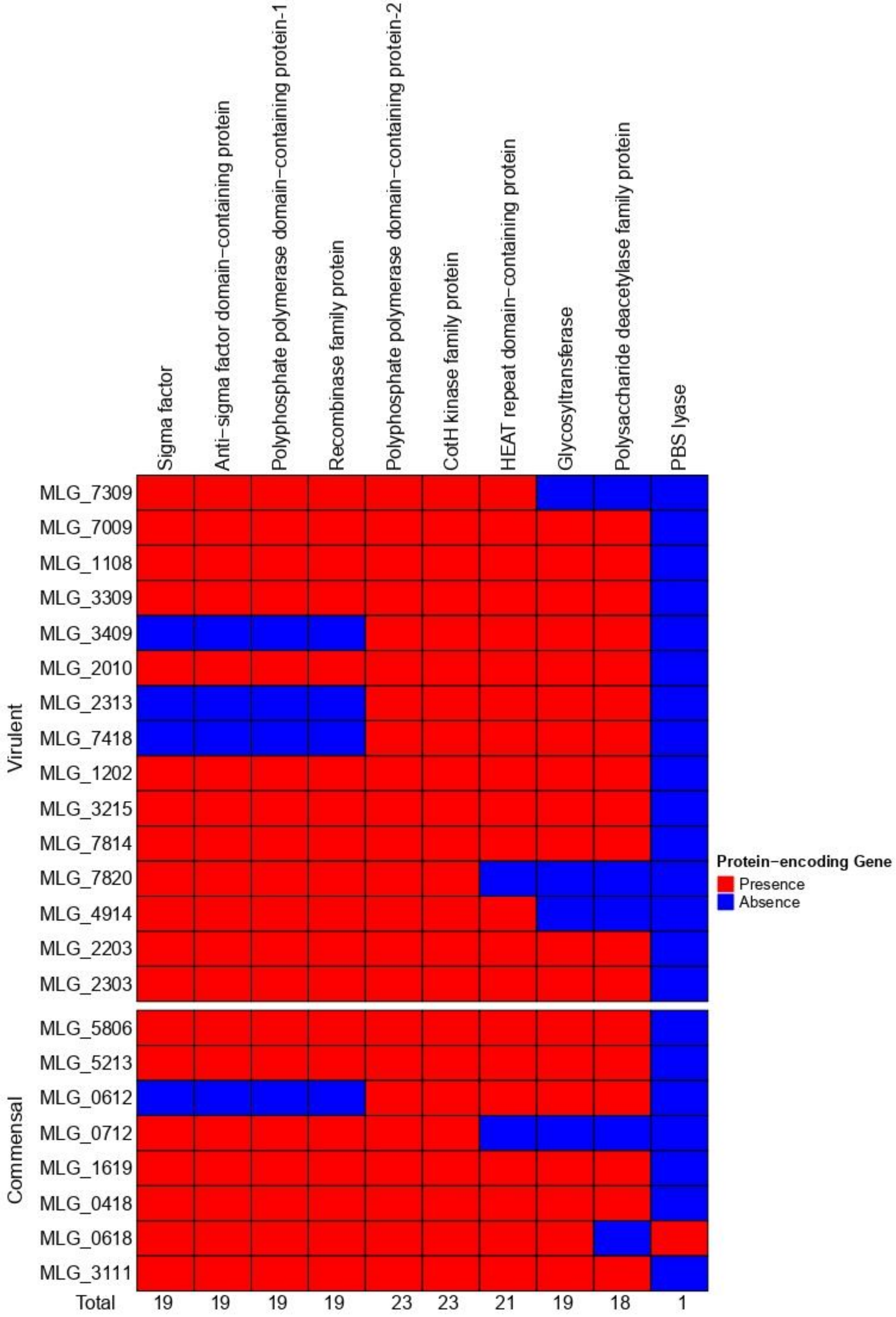
Sigma factor
Anti−sigma factor domain−containing protein
Polyphosphate polymerase domain−containing protein-1
Recombinase family protein
Polyphosphate polymerase domain−containing protein-2
CotH kinase family protein
HEAT repeat domain−containing protein
Glycosyltransferase
Polysaccharide deacetylase family protein
PBS lyase
Virulent
MLG_7309
MLG_7009
MLG_1108
MLG_3309
MLG_3409
MLG_2010
MLG_2313
MLG_7418
MLG_1202
MLG_3215
MLG_7814
MLG_7820
MLG_4914
MLG_2203
MLG_2303
Commensal
MLG_5806
MLG_5213
MLG_0612
MLG_0712
MLG_1619
MLG_0418
MLG_0618
MLG_3111
Total 19 19 19 19 23 23 21 19 18 1
Protein−encoding Gene
Presence
Absence

(C)

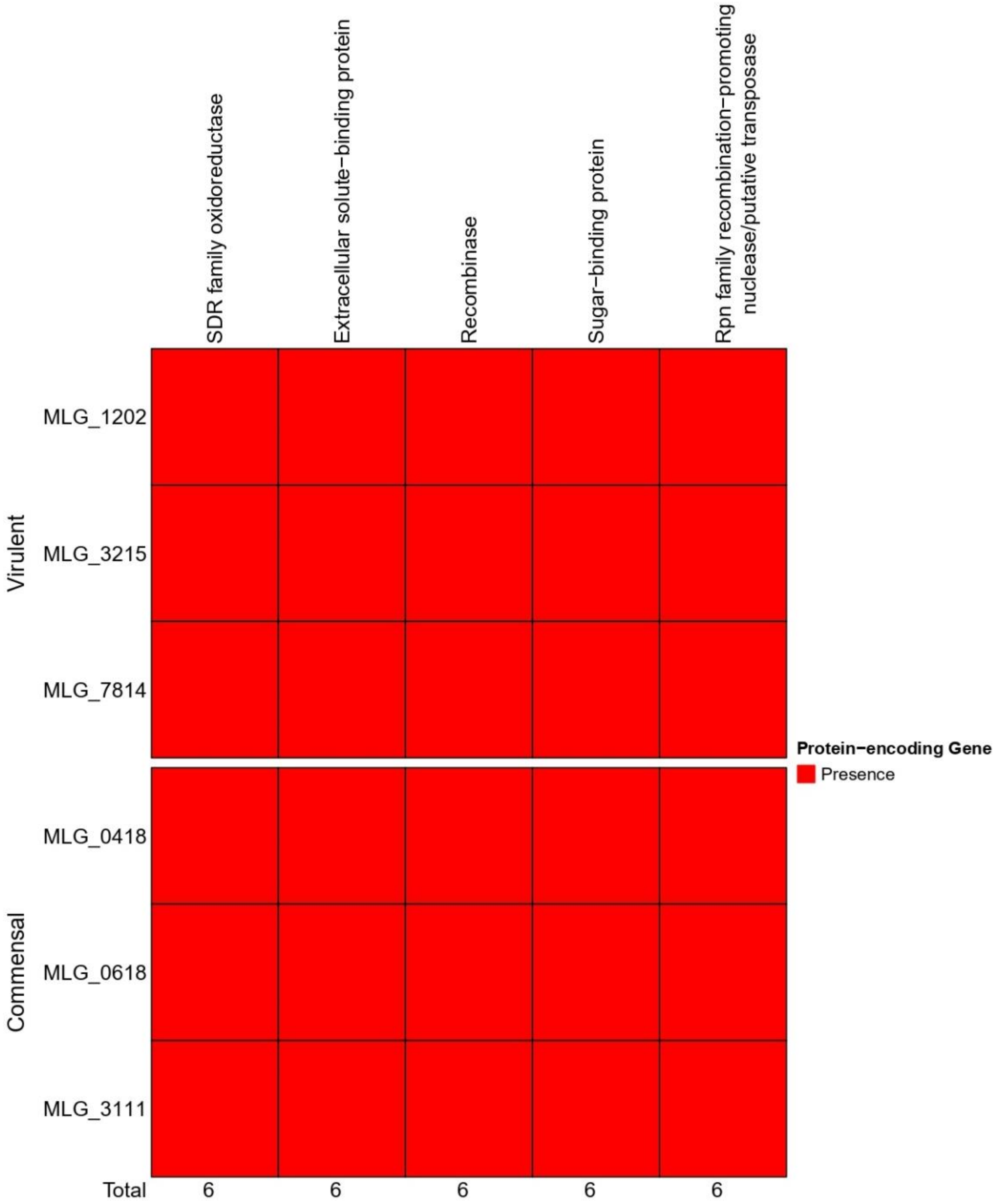

Figure S2. Heat maps presenting the identified genes within (A) NELoc-1, (B) NELoc-2, and (C) NELoc-3 and their distribution among *C. perfringens* strains using "ComplexHeatmap" in the R package. The presence of genes is indicated by cell colours: red (presence) and blue (absence).

(A)

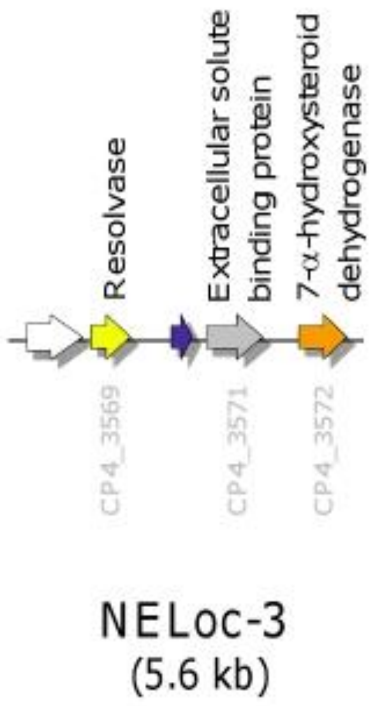

(B)

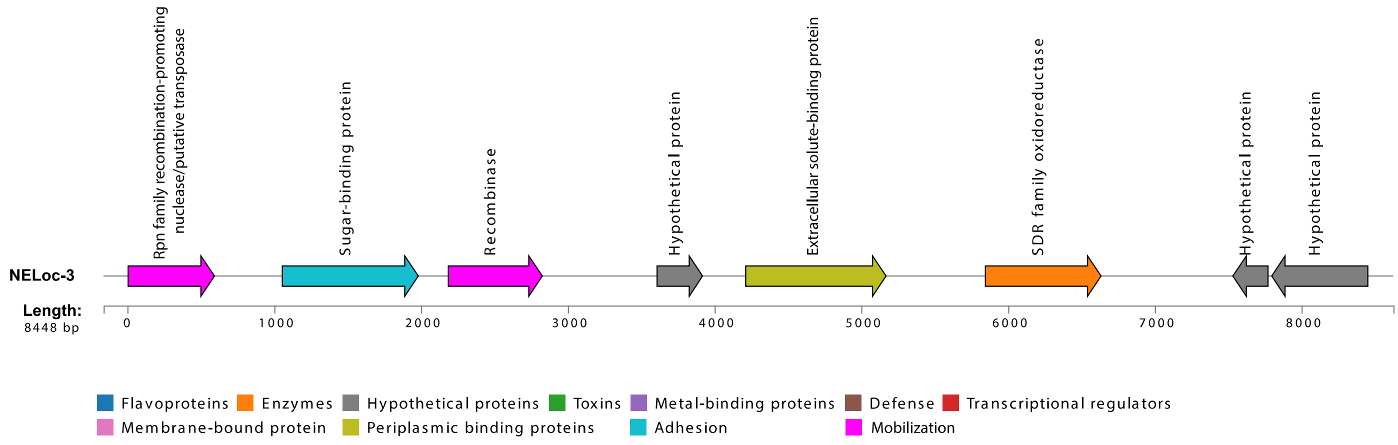

Figure S3. Comparison between (A) the previous findings by Lepp et al. [14] and (B) our results regarding NEloc-3.
